# Ventral hernia repair with enhanced-view totally extraperitoneal technique after a massive weight loss by laparoscopic sleeve gastrectomy

**DOI:** 10.1186/s40792-023-01610-1

**Published:** 2023-02-20

**Authors:** Manabu Amiki, Yasuhiro Ishiyama, Ichitaro Mochizuki, Kazuhiro Narita, Manabu Goto, Koji Sekikawa

**Affiliations:** Kawasaki Saiwai Hospital, 31-27 Omiya-Cho, Saiwai-Ku, Kawasaki City, Kanagawa 212-0014 Japan

**Keywords:** Ventral hernia, eTEP, Laparoscopic sleeve gastrectomy, Metabolic and bariatric surgery

## Abstract

**Background:**

Ventral hernia repair (VHR) for obese patients is often associated with an increased risk of postoperative complications and hernia recurrences. Achieving preoperative weight loss is ideal before VHR; however, it is difficult to attain with medical treatment. Metabolic and bariatric surgery (MBS) offers the most effective and durable treatment for obesity. Therefore, massive weight loss occurring after MBS will improve the outcome of VHR.

**Case presentation:**

A 49-year-old man (122.9 kg, BMI 39.1 kg/m^2^) presented to our hospital wishing to undergo laparoscopic sleeve gastrectomy and VHR. Physical examination revealed a tennis ball-sized lower midline defect. Computed tomography (CT) scans revealed a hernia orifice 5 cm in width and 10 cm in height. As the hernia orifice was large, mesh reinforcement was essential. We planned for him to undergo VHR after massive weight loss was achieved by MBS. VHR was performed using the enhanced-view totally extraperitoneal (eTEP) technique after weight loss of 38 kg was achieved 9 months following laparoscopic sleeve gastrectomy. His postoperative course was uneventful, and neither recurrence nor seroma was observed at 1 year follow-up.

**Conclusions:**

eTEP repair of a ventral hernia after massive weight loss following MBS would appear to be the best combination treatment for obese patients with ventral hernias. However, long-term follow-up is necessary to establish its safety and efficacy.

## Background

Obesity is associated with a high risk for the development of ventral hernias [1], the prevalence of which was reported to reach 8% in patients undergoing laparoscopic Roux-en-Y gastric bypass [2]. In addition, obesity increases the risk of postoperative complications and recurrences after ventral hernia repair (VHR) [3, 4]. Therefore, obese patients who plan to undergo VHR are strongly encouraged to lose weight preoperatively; however, this is very difficult to achieve with currently available medical treatment [5] other than metabolic and bariatric surgery (MBS). MBS offers the best option for treating obesity and its associated comorbidities [5]. Massive weight loss after MBS facilitates VHR and reduces postoperative complications and recurrences. Here, we report a case of an obese patient with a ventral hernia repaired using the enhanced-view totally extraperitoneal (eTEP) technique 9 months after laparoscopic sleeve gastrectomy (LSG).

## Case presentation

**Fig. 1:**
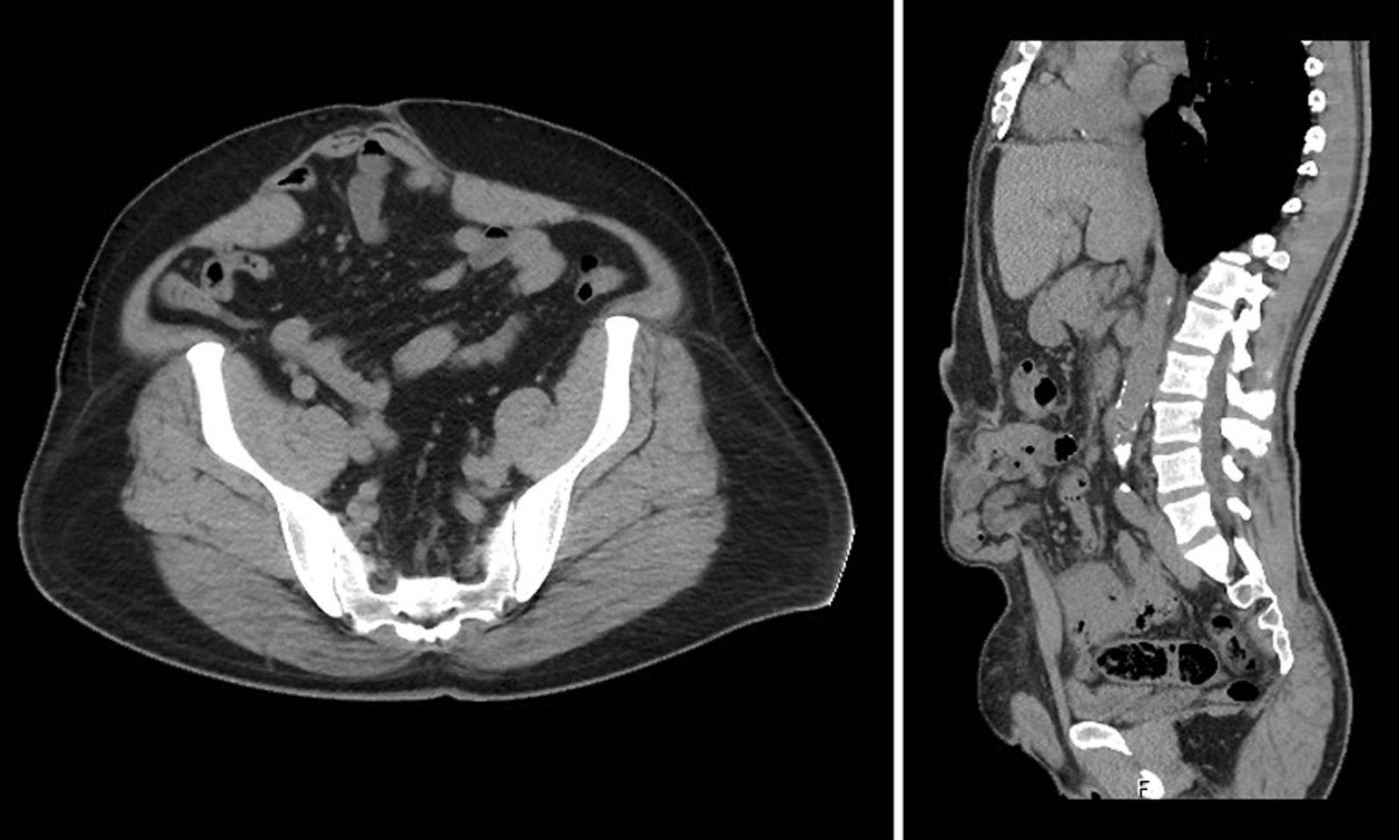
Abdominal CT shows an incisional hernia orifice in the infra-umbilical region (M3, M4)

A 49-year-old man (122.9 kg, BMI 39.1 kg/m^2^) presented to our hospital wishing to undergo LSG and VHR. His medical history included diabetes mellitus, hypertension, and hyperlipidemia. He had undergone urgent surgery for an umbilical hernia with incarcerated small bowel 18 months ago. The surgery included small bowel resection and direct closure of the defect without mesh reinforcement. Six months later, the umbilical hernia recurred. Physical examination revealed a tennis ball-sized lower midline defect. Computed tomography (CT) scans revealed a hernia orifice 5 cm in width and 10 cm in height in the infra-umbilical region (Fig. 1). As mesh reinforcement was essential to repair the ventral hernia, we planned for him to undergo VHR after LSG.

LSG was performed using a standardized surgical technique with a 37.5 Fr bougie. Intraoperative findings revealed adhesion between the omentum and the hernia sac, which was not dissected during LSG. His postoperative course was uneventful, and after 9 months, he showed satisfactory weight loss (84.2 kg, BMI 26.8 kg/m^2^) and his comorbidities such as diabetes mellitus, hypertension, and hyperlipidemia were in remission. We then performed VHR using the eTEP technique.

**Fig. 2:**
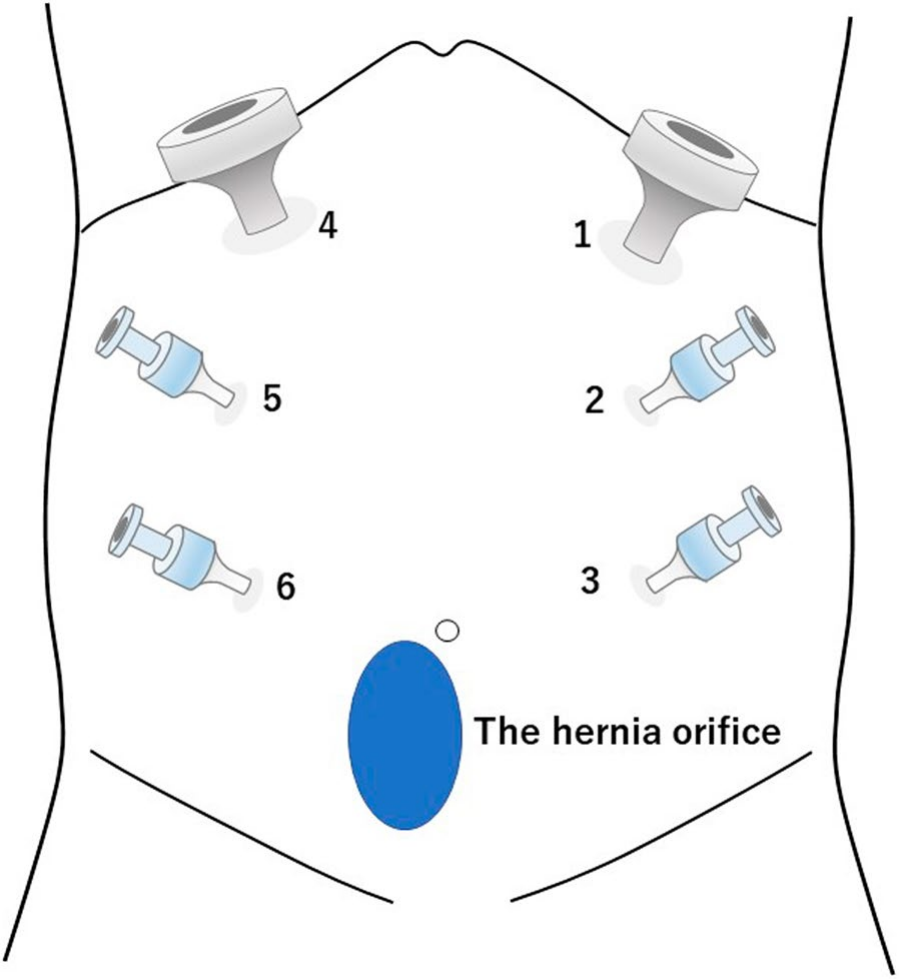
Port positions

**Fig. 3:**
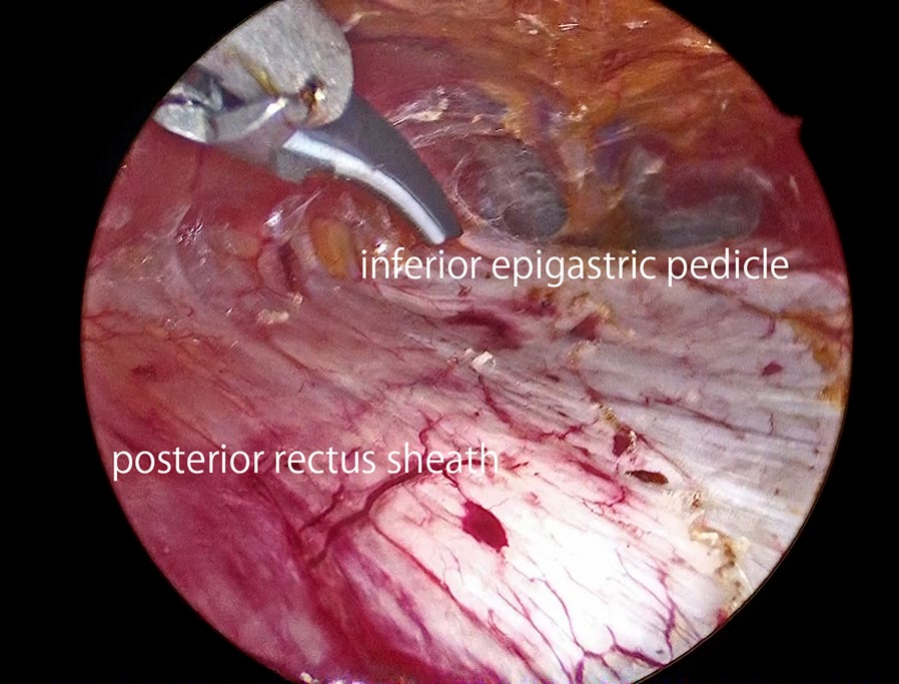
The left retrorectus space was developed

**Fig. 4:**
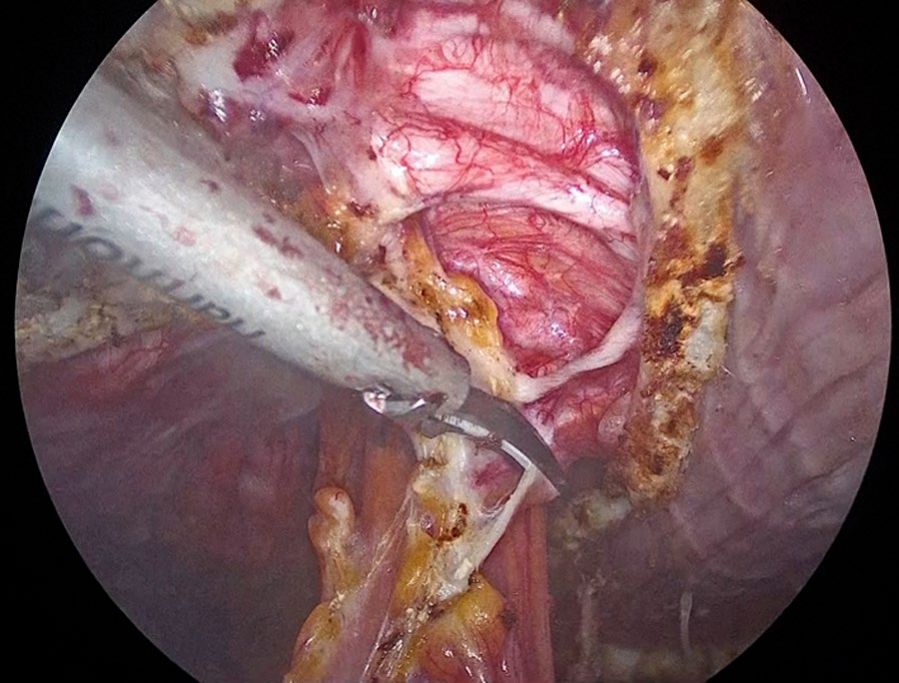
Intraperitoneal dissection and repositioning of hernia contents

**Fig. 5:**
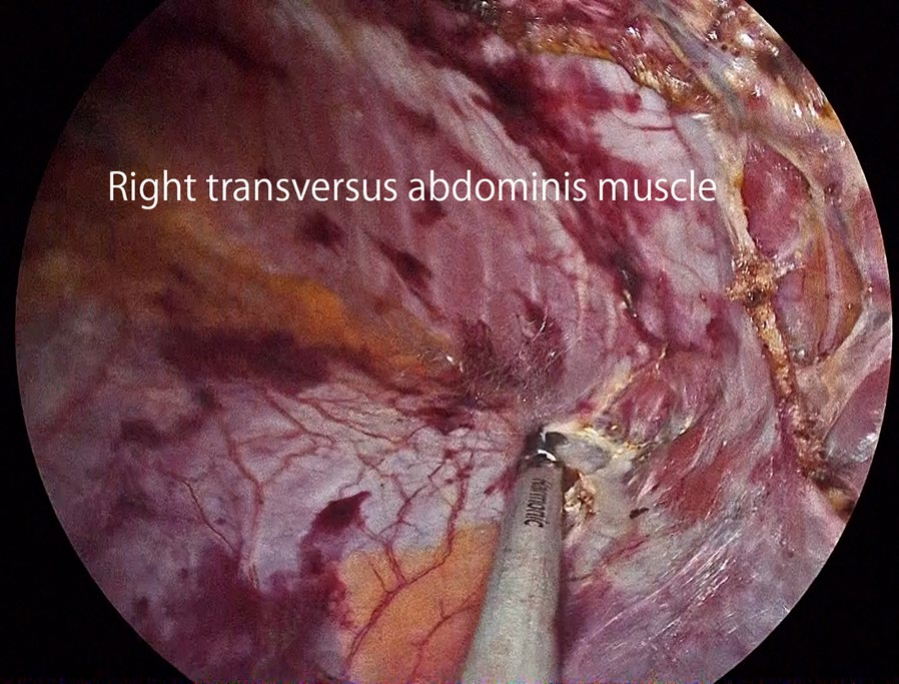
Bilateral transversus abdominis muscle release was performed

**Fig. 6:**
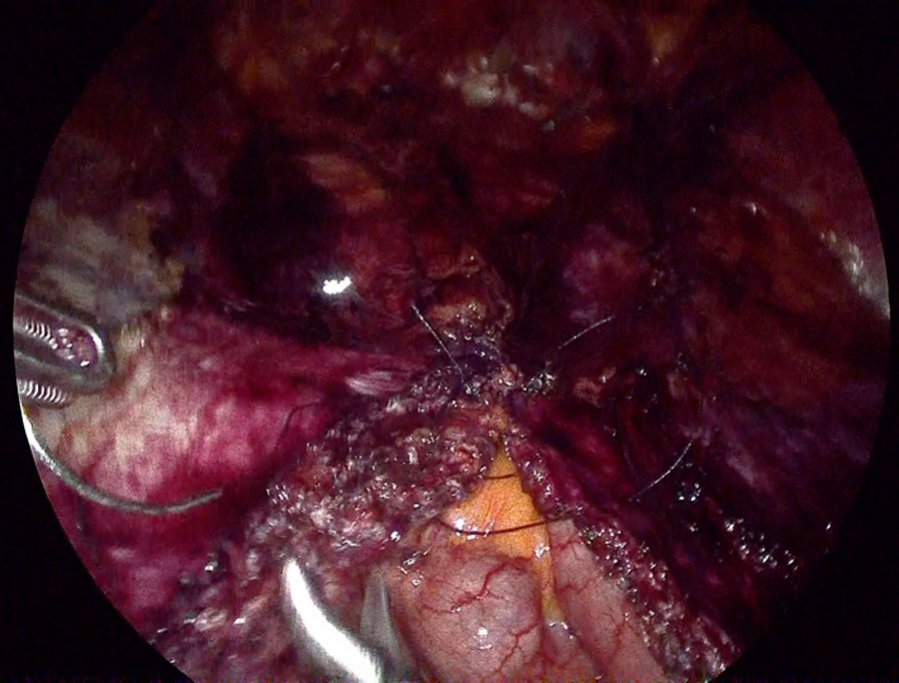
The posterior layer defect was closed with 3-0 multifilament suture material

**Fig. 7:**
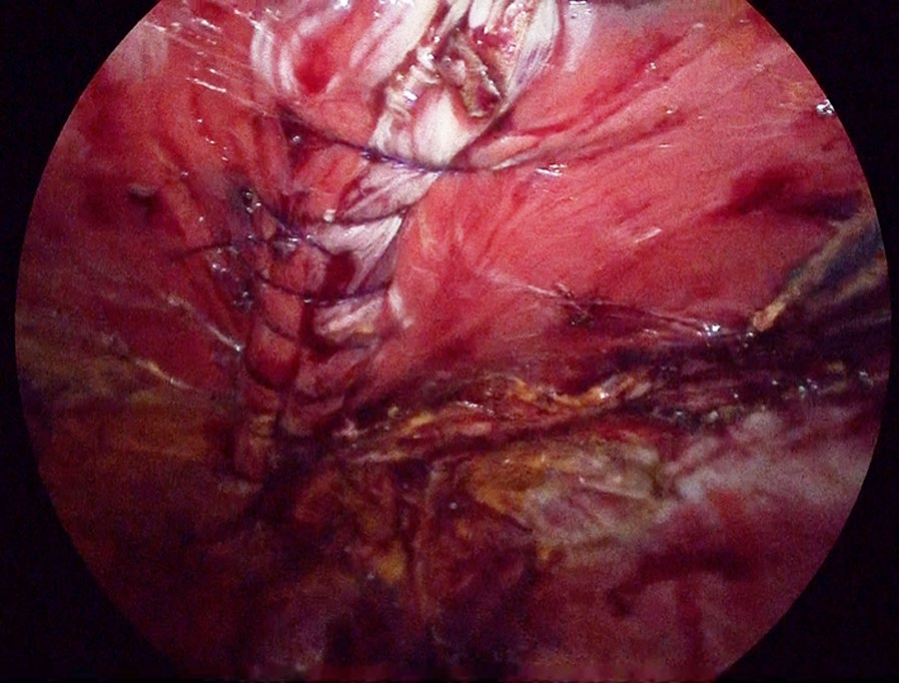
The linea alba was restored with 1-0 barbed sutures

**Fig. 8:**
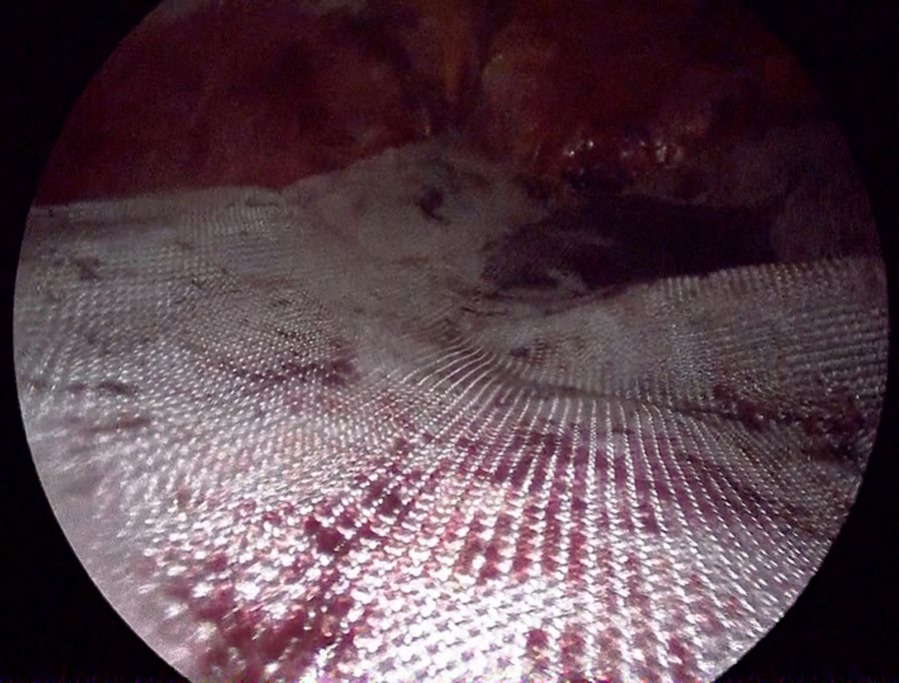
Medium-weight small-pore polypropylene mesh of 26 × 35.5 cm was positioned

**Fig. 9:**
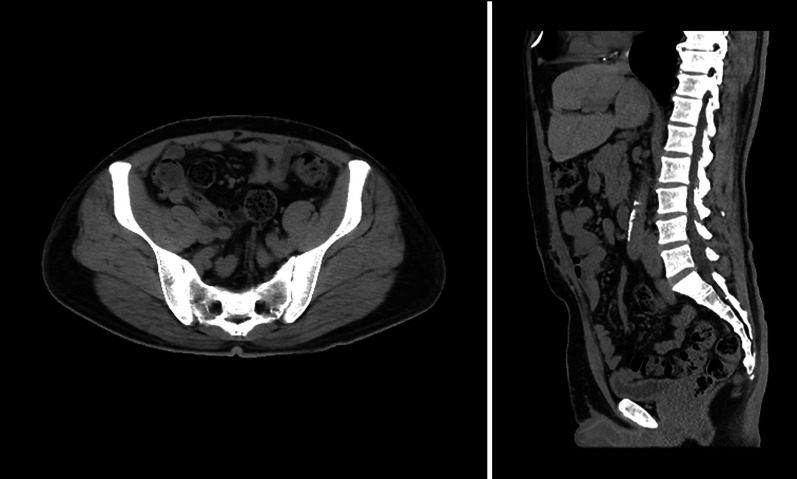
CT scan images at 4 months follow-up

After induction of general anesthesia and intubation, he was positioned with the bilateral upper extremities tucked at his sides. Figure 2 shows the port placement in this case. A 1.5-cm skin incision was made just below the left costal margin, and the anterior rectus sheath was identified and incised sharply. A 12-mm trocar was inserted posterior to the rectus abdominis muscle, and the left retrorectus space was developed (Fig. 3) followed by insertion of two 5-mm trocars at the port 2 and port 3 positions medial to the linea semilunaris. The left posterior rectus sheath was incised close to the linea alba, and then the right posterior rectus sheath was opened over the falciform ligament. After the preperitoneal and two retrorectus spaces were connected, a 12-mm trocar was inserted at port 4, and lateral dissection of the right posterior rectus sheath was done. Two 5-mm trocars were inserted at the port 5 and port 6 positions. The sac was opened, and intraabdominal adhesions were dissected (Fig. 4). Bilateral transversus abdominis muscle release was performed (Fig. 5). The posterior layer defect was closed with 3-0 multifilament suture material (Fig. 6). Then, the linea alba was restored with 1-0 barbed sutures (Fig. 7). BARD™ Mesh (Davol Inc., Warwick, RI, USA 02,886), a medium-weight small-pore (0.44 mm) polypropylene mesh of 26 cm width × 35.5 cm height, was positioned to cover the dissected area with no fixation (Fig. 8). A 19 Fr drain was placed over the mesh. The operating time was 452 min, and the amount of blood loss was nearly 0 g. The patient’s postoperative course was uneventful, the drain was removed 4 days after the surgery, and he was discharged on the same day. A CT scan 4 months after eTEP repair did not show recurrence or seroma (Fig. 9). At 1-year follow-up, he was doing well.

## Discussion

The appropriate management of obese patients with ventral hernias is still controversial. If obese patients become bariatric candidates and agree to undergo MBS, massive weight loss will be guaranteed. In this situation, the main issue is mainly focused on the timing of VHR, which is performed during or after MBS. Another issue is what kind of MBS and VHR is optimal for this subgroup of patients.

VHR performed during MBS is a definite benefit for patients as they do not have to undergo two separate surgeries. In addition, complications from the untreated hernia, such as bowel obstruction, may be avoided. Smaller hernias can be closed with simple suturing during MBS. However, mesh repair should be performed for most bariatric candidates needing VHR because mesh repair is recommended even for an umbilical hernia > 1 cm [6]. It is controversial whether to place mesh in clean-contaminated fields. Some authors have insisted on the safety of placing prosthetic mesh during MBS [2, 7–11], but limited data were presented.

Several studies have reported that VHR after MBS could reduce wound complications and recurrence rates [12–19]. Consequently, the current 2022 ASMBS and IFSO guidelines recommend two staged repairs [20]. Ventral hernias in obese patients tend to be large, which adds to the complexity of repair. Massive weight loss after MBS will increase the chances of a successful repair in patients with large or complex hernias. However, Eid et al. reported that deferring VHR after MBS was associated with a high rate of small bowel obstruction [7]. Therefore, the waiting period should not be unnecessarily prolonged. As weight loss generally stabilizes 9–12 months after MBS, VHR should be considered at that time [21].

The choice of optimal MBS for this subgroup of patients is another controversial issue. Ventral hernias can affect MBS, either by preventing adequate visualization of the operative field or by limiting the mobility of the small bowel required to restore gastrointestinal continuity. In this situation, laparoscopic Roux-en-Y gastric bypass is often not feasible. As LSG avoids small bowel adhesiolysis and thus prevents adhesiolysis-related morbidity, we consider LSG to be the preferred technique.

The open sublay technique (Rives–Stoppa) and laparoscopic intraperitoneal onlay mesh (IPOM) technique are commonly used in VHR; however, these procedures have their specific disadvantages [22, 23]. Rives–Stoppa has a higher rate of surgical site infection than laparoscopic VHR [22]. Furthermore, patients following MBS have a higher risk of surgical site occurrence after surgery requiring a large skin incision [24]. These findings indicate that open VHR, such as Rives–Stoppa, should be avoided, especially after MBS when laparoscopic VHR is available. eTEP repair was introduced in 2018 [25]. This technique can be performed laparoscopically and allows a surgeon to place a large flat synthetic mesh in the retrorectus plane. When comparing IPOM and eTEP, the latter is better in terms of less early postoperative pain and earlier return to activities [26]. Therefore, we considered that eTEP would be the best method for VHR after MBS. However, eTEP is technically challenging and prolongs operative time, which increases the potential perioperative risk for obese patients. Furthermore, entering the abdominal cavity for MBS makes it very difficult to maintain insufflation of the retromuscular space unless all ports for MBS are placed lateral to the semilunar line. This would indicate that eTEP access repair during MBS is technically challenging. Therefore, eTEP repair should be performed after massive weight loss is achieved with MBS.

## Conclusion

To our knowledge, this is the first case report describing the use of the eTEP technique after LSG. Although long-term follow-up is necessary to establish its safety and efficacy, eTEP repair after massive weight loss achieved with MBS may be the best treatment for obese patients with ventral hernia.

## Data Availability

All data generated or analyzed during this study are included in this published article.

## Abbreviations

VHR: Ventral hernia repair
MBS: Metabolic and bariatric surgery
eTEP: Enhanced-view totally extraperitoneal
LSG: Laparoscopic sleeve gastrectomy
CT: Computed tomography
IPOM: Intraperitoneal onlay mesh
